# Effect of bone marrow mesenchymal stem cell transplantation on acute hepatic failure in rats

**DOI:** 10.3892/etm.2014.1848

**Published:** 2014-07-16

**Authors:** SHUFANG YUAN, TAO JIANG, RONGJIONG ZHENG, LIHUA SUN, GUIQIU CAO, YUEXIN ZHANG

**Affiliations:** 1 Department of Infectious Diseases, The First Affiliated Hospital of Xinjiang Medical University, Urumqi, Xinjiang 830011, P.R. China; 2 The Fifth Affiliated Hospital of Xinjiang Medical University, Urumqi, Xinjiang 830011, P.R. China; 3 Key Laboratory of Xinjiang Medical Animal Model Research, Urumqi, Xinjiang 830011, P.R. China; 4 State Key Laboratory Incubation Base of Xinjiang Major Diseases Research, The First Affiliated Hospital of Xinjiang Medical University, Urumqi, Xinjiang 830054, P.R. China

**Keywords:** acute hepatic failure, bone mesenchymal stem cells, cluster of differentiation 163, interleukin 10, rat model

## Abstract

The aim of the present study was to investigate the effectiveness of bone marrow mesenchymal stem cell (BMSC) transplantation in the treatment of acute hepatic failure (AHF) in rats. BMSCs were isolated from rat bone marrow, cultured and analyzed by flow cytometry. Following BMSC transplantation into rats with AHF, the levels of alanine aminotransferase (ALT), aspartate aminotransferase (AST), albumin (ALB), direct bilirubin (DBIL) and indirect bilirubin (IBIL) in the serum were measured using an automatic biochemical analyzer. Hematoxylin and eosin (H&E) staining and a terminal deoxynucleotidyl transferase dUTP nick end labeling (TUNEL) assay were performed to analyze the pathological changes and apoptosis rate. Levels of cluster of differentiation (CD)163 and interleukin (IL)-10 in the serum and liver tissue were detected by an enzyme-linked immunosorbent assay (ELISA) assay and western blot analysis. Compared with the levels in the control group, the serum levels of ALT, AST, DBIL, IBIL, CD163 and IL-10 in the BMSC transplantation groups were significantly lower at 120 and 168 h, while the serum levels of ALB were significantly higher at 168 h after BMSC transplantation. The pathological features of liver failure were alleviated by BMSC transplantation. The expression levels of CD163 and IL-10 in the liver tissue were also significantly decreased following transplantation. The results indicate that BMSCs have a therapeutic effect on AHF in rats, and CD163 and IL-10 may be used as sensitive serum prognosis indicators in the early assessment of patients following liver transplantation.

## Introduction

Acute hepatic failure (AHF), a clinical syndrome caused by multiple factors, is characterized by severe liver cell damage. In patients with AHF, the functions of synthesis, metabolism, transport and excretion in the liver are severely impaired, ultimately leading to multiple organ failure and mortality. Due to its complex clinical symptoms and high mortality rate, the treatment of AHF is challenging for the medical community (1,2). In addition to orthotopic liver transplantation (OLT), stem cell transplantation has become an effective means for the treatment of liver failure in recent years (3–6).

The pathogenesis of AHF is associated with the immune response and inflammatory reactions in the body. Previous studies have revealed that macrophage activation and the secretion of multiple cytokines plays an important role in the pathogenesis of AHF (7,8). Hepatic apoptosis and necrosis mediated by inflammatory cytokines are important factors in the development of AHF (9,10). Furthermore, the inflammatory response in the body may be regulated by anti-inflammatory factors. Cluster of differentiation (CD)163 and interleukin (IL)-10 play important roles in anti-inflammatory reactions. CD163 is specifically expressed on the membranes of monocyte and macrophage cells. Its anti-inflammatory and anti-oxidative effects play a critical role in the occurrence and development of liver failure (11,12). IL-10 upregulates the expression of CD163 in monocytes and reduces the inflammatory response of hepatic cells and the adhesion of neutrophils to sinusoidal endothelial cells, thereby alleviating liver damage (13–15). Parekkadan *et al* (16) demonstrated that cytokines derived from bone marrow mesenchymal stem cells (BMSCs) may prevent the necrosis of hepatic cells and improve the survival rate of patients with fulminant hepatic failure. Kuo *et al* (17) induced lethal fulminant hepatic failure in non-obese diabetic severe combined immunodeficient mice with CCl_4_. Mesenchymal stem cell-derived hepatocytes and BMSCs were intrasplenically or intravenously transplanted at different doses. The study revealed that BMSC transplantation was able to effectively prevent experimental liver failure.

In the present study, an AHF model was established in rats. The therapeutic effect of BMSC transplantation on AHF was investigated. Liver function was assessed by detecting the levels of alanine aminotransferase (ALT), aspartate aminotransferase (AST), albumin (ALB), direct bilirubin (DBIL) and indirect bilirubin (IBIL) in the serum. Pathological liver damage was revealed by hematoxylin and eosin (H&E) staining. The dynamic changes in the levels of CD163 and IL-10 in the serum and liver tissue were also measured. Furthermore, the cell apoptosis rate in the liver was observed by terminal deoxynucleotidyl transferase dUTP nick end labeling (TUNEL) staining.

## Materials and methods

### Animals

Sprague-Dawley (SD) rats [healthy males; weighing ~250–300 g; specific pathogen-free (SPF)-class] were provided by the Animal Center of the Centers for Disease Control in Xinjiang (License no. SCXK Xin 2003-0002). The rats were kept under standard conditions. All animal experiments were performed with the approval of the Medical Ethics Committee of Xinjiang Medical University (Urumqi, China).

### Separation, culture and identification of rat BMSCs

BMSCs were isolated from the bone marrow of SD rats. Briefly, 50,000 units heparin was injected intraperitoneally into each rat. Following injection for 15 min, the rats were sacrificed and whole bone marrow was collected from the bilateral tibia. Cells from the whole bone marrow were seeded in a 75-cm^2^ Petri dish at a concentration of 1×10^9^ cells/l. Following incubation for 7–10 days, colonies were established. Cells from the colonies were collected and plated into dishes. After achieving 70–80% confluence, adherent cells were trypsinized using 0.25% (w/v) trypsin/ethylenediaminetetraacetic acid (EDTA; Invitrogen Life Technologies, Carlsbad, CA, USA) and replated at a dilution of 1:3. At the third passage, BMSCs were identified and cultured for future experiments.

BMSCs were identified by flow cytometric analysis (Gallios Flow Cytometer, Beckman Coulter Inc., Brea, CA, USA). Briefly, the cells were collected, washed with phosphate-buffered saline (PBS) and resuspended at a concentration of 1×10^9^/l. The single cell suspension was incubated with fluorescein isothiocyanate (FITC)-labeled mouse anti-CD45, CD29, CD11 and CD90 antibodies (BioLegend, Inc., San Diego, CA, USA). Following incubation at room temperature for 15 min, the cells were washed twice with PBS and analyzed by flow cytometry.

### Animal model and BMSC transplantation

The model of AHF was induced by the intraperitoneal injection of 10% D-galactosamine (1.4 g/kg, administered twice in 12 h) and 0.005% lipopolysaccharide (LPS; 20 μg/kg, administered once in 12 h)(both from Sigma, St. Louis, MO, USA). Following model establishment, the rats were fasted for another 12 h prior to BMSC transplantation.

Isolated BMSCs were transplanted through portal and tail vein injection. A total of 12 SD rats without any prior treatment were used as the normal group. A total of 48 SD rats were used for the animal model establishment. These were randomly divided into the control group with liver failure (n=16), the tail vein transplantation group (n=16) and the portal vein transplantation group (n=16). For the control group, rats were administered the same volume of saline by intraperitoneal injection. For the tail vein group, rats were injected with BMSCs (1.4×10^7^ cells/kg) through the tail vein. For the portal vein group, the rats were anaesthetized and the abdominal cavity was exposed. BMSCs (1.4×10^7^ cells/kg) were injected through the portal vein. At 24, 120 and 168 h following transplantation, blood samples and liver tissue were collected from the rats for further analysis. Liver tissues were fixed with 4% paraformaldehyde.

### Serum analysis

Blood samples were centrifuged and the serum was isolated. The levels of ALT, AST ALB, DBIL and IBIL in the serum were measured using an automatic biochemical analyzer (UniCel DxC 800; Beckman Coulter, Miami, FL, USA). The levels of CD163 and IL-10 were detected using an enzyme-linked immunosorbent assay (ELISA) according to the manufacturers’ instructions. The anti-CD163 antibody was purchased from the Shanghai Westang Bio-Tech, Co. Ltd Co., Ltd. (Shanghai, China), and the anti-IL-10 antibody was purchased from eBioscience, Inc. (San Diego, CA, USA).

### H&E staining

Liver tissues were embedded in paraffin, cut into 4-μm sections and stained with H&E. For pathological observation under a light microscope (Olympus BX50, Olympus, Tokyo, Japan), five views at low magnification (x200) and five views at high magnification (x400) were randomly selected from each slide.

### TUNEL assay

The cell apoptosis rate in the liver was detected by a TUNEL assay kit (Roche Applied Science, Rotkreuz, Switzerland). Briefly, paraffin sections were dewaxed, hydrated and rinsed with PBS. Subsequently, H_2_O_2_ in methanol (3 ml/l) solution was added to block endogenous peroxidase activity. The liver sections were permeabilized by adding permeabilization solution (1 g/l Triton X-100 dissolved in 0.1% sodium citrate). Following permeabilization, the TUNEL reaction buffer and Converter-POD were added. Finally, the liver sections were incubated with 3,3′-diaminobenzidine (DAB) for chromogenic reaction. Sections were counter-stained with hematoxylin to identify the background TUNEL-negative cells. Hepatic apoptosis was observed under a microscope (Olympus BX50, Olympus). For the negative control, phosphate buffer instead of the primary antibody was added. The apoptotic cells were characterized by brown particles confined within the nucleus. Five fields at high magnification (x400) were randomly selected from each slice. The percentage of apoptotic cells was calculated as the ratio of the TUNEL positive cell number to the total liver cell number per field.

### Western blot analysis

Protein samples were extracted from the liver tissue and separated with 10% polyacrylamide gel electrophoresis. The proteins were transferred onto membranes. Following blocking, the membranes were incubated with antibodies. The primary antibodies were mouse anti-CD163 (1:200; sc-58965; Santa Cruz Biotechnology, Inc., Santa Cruz, CA, USA), mouse anti-IL-10 (1:250; ab25073; Abcam, Cambridge, MA, USA) and glyceraldehyde 3-phosphate dehydrogenase (GAPDH; 1:1,000; sc-25778; Santa Cruz Biotechnology). The secondary antibody was goat anti-mouse immunoglobulin G (IgG; Thermo Scientific, Pierce Biotechnology, Inc., Rockford, IL, USA). Finally, the membranes were developed using enhanced chemiluminescence (ECL; Amersham Pharmacia Biotech, Piscataway, NJ, USA). The gray value was measured using Quantity One software (Bio-Rad, Hercules, CA, USA). The gray value of GAPDH was used as an internal control.

### Statistical analysis

Data were analyzed using SPSS software, version 18.0 (SPSS, Inc., Chicago, IL, USA). Data are expressed as mean ± standard deviation. Differences between the groups were compared using randomized block analysis of variance (ANOVA). Differences between measurements were analyzed using ANOVA following rank conversion. Correlation analysis was conducted using Spearman’s correlation analysis. P<0.05 was considered to indicate a statistically significant difference.

## Results

### Levels of ALT, AST, DBIL, IBIL, CD163 and IL-10 in the serum decrease while the level of ALB increases following BMSC transplantation

**Figure 1:**
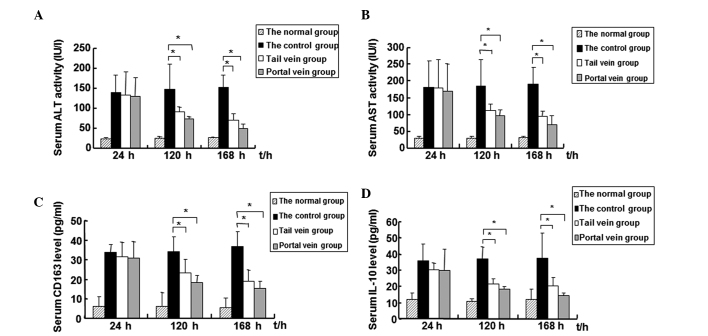
Levels of alanine aminotransferase (ALT), aspartate aminotransferase (AST), cluster of differentiation (CD)163 and interleukin (IL)-10 in the serum. At 24, 120 and 168 h following bone marrow mesenchymal stem cell (BMSC) transplantation, serum was collected from the normal, control, tail vein and portal vein group rats. Levels of (A) ALT and (B) AST in the serum as detected by an automatic biochemical analyzer. Levels of (C) CD163 and (D) IL-10 in the serum as detected by an enzyme-linked immunosorbent assay (ELISA). Experiments were performed three times. Data are expressed as mean ±standard deviation. ^*^P<0.05, control vs. the tail and portal vein groups.

Levels of ALT, AST, ALB, DBIL and IBIL in the serum are biomarkers of liver injury and reflect liver function to a certain extent. To evaluate liver function following BMSC transplantation, the blood samples of rats were collected at 24, 120 and 168 h following transplantation. The levels of ALT, AST, ALB, DBIL and IBIL in the serum were measured using an automatic biochemical analyzer. The results are shown in Fig. 1A and B, and Tables I–III. As shown in Fig. 1A and B, the levels of ALT and AST in the tail and portal vein groups gradually decreased over the treatment time period. At 168 h (1 week) following transplantation, the ALT and AST levels had decreased to their lowest level. The levels of ALT and AST in the AHF control group remained at a high level throughout the experimental period. Statistically, there were no significant differences in the levels of ALT and AST among the three groups at 24 h following BMSC transplantation. At 120 and 168 h following transplantation, the levels of ALT and AST in the tail and portal vein groups were significantly lower compared with those in the control group (P<0.05). Notably, the levels of ALT and AST in the portal vein group were slightly lower than those in the tail vein group at the three time points; however, the differences between the two groups were not statistically significant. Similarly, compared with the levels in the control group, the serum levels of DBIL and IBIL in the tail and portal vein groups were significantly decreased at 120 and 168 h (P<0.05; Table II). However, the tail and portal vein groups had significantly higher levels of ALB at 168 h compared with the control group (P<0.05; Table III). Furthermore, the differences in the serum levels of DBIL, IBIL and ALB were not significant between the tail and portal vein groups. These results suggest that liver injury was reduced and liver function was improved following BMSC transplantation.

Changes in the levels of the inflammatory factors (CD163 and IL-10) in the serum were also detected by an ELISA assay. In the control group, the AHF model rats did not undergo stem cell transplantation. Results are shown in Fig. 1C and D, and Table IV. As shown in Fig. 1C and D, the serum levels of CD163 and IL-10 in the control group increased over time, indicating a deterioration in liver function. At 24 h following BMSC transplantation, the levels of CD163 and IL-10 in the tail and portal vein groups were not observed to be significantly different when compared with the control group. However, at 120 and 168 h following BMSC transplantation, the levels of CD163 and IL-10 in the tail and portal vein groups gradually decreased and were significantly lower than those in the control group (P<0.01). Similarly, the levels of CD163 and IL-10 in the portal vein group were lower than those in the tail vein group. However, these differences were not found to be statistically significant.

A correlation analysis was carried out between the levels of CDl63 and IL-10 and the levels of ALT and AST at 168 h. Significant correlations were identified between CD163 and ALT (r=0.460, P=0.048) and AST (r=0.492, P=0.033). There were also significant correlations between IL-10 and ALT (r=0.530, P=0.02) and AST (r=0.618, P=0.005). Furthermore, the levels of CD163 were positively correlated with the levels of IL-10 (r=0.733, P=0.001).

### BMSC transplantation alleviates the pathological features of liver damage in an AHF rat model

**Figure 2:**
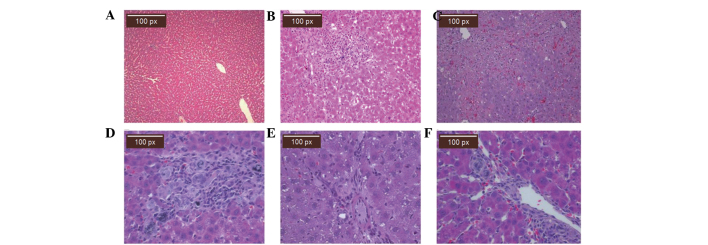
Pathological features of liver tissue by hematoxylin and eosin (H&E) staining. At 24, 120 and 168 h following bone marrow mesenchymal stem cell (BMSC) transplantation, liver tissue was collected from healthy, control, tail vein and portal vein group rats. The tissues were stained with H&E. (A) Histological features of healthy rat liver tissue (magnification, ×100). Pathological features of liver tissue in (B) the control group at 24 h (magnification, ×200), (C) the control group at 168 h (magnification, ×200), (D) the portal vein group at 120 h following BMSC transplantation (magnification, ×400), (E) the portal vein group at 168 h following BMSC transplantation (magnification, ×400) and (F) the tail vein group at 168 h following BMSC transplantation (magnification, ×400).

To observe the pathological features following BMSC transplantation, liver tissue was collected and stained with H&E. Fig. 2A shows the histological features of healthy rat liver tissue. In the normal liver tissue, the structure of the hepatic lobule was intact. Hepatocytes of similar cellular sizes were arranged in cords within the hepatic lobule. These cords radiated outwards from the central veins. The pathological features of the liver tissue from rats with AHF are shown in Fig. 2B and C. In Fig. 2B, the pathological features of the liver tissue at 24 h following the injection of D-galactosamine in the control group are shown. The hepatic lobule had lost its normal structure and the hepatocytes were swollen and degenerated. There was inflammatory cell infiltration in the portal area. In Fig. 2C, the pathological features of the liver tissue at 168 h following the injection of D-galactosamine in the control group are shown. Hepatocytes revealed diffuse and confluent necrosis with bridging. Significant proliferation of inflammatory cells was observed in the portal area. The pathological features of the liver tissue with BMSC transplantation are shown in Fig. 2D (120 h following BMSC transplantation through the portal vein), Fig. 2E (168 h following BMSC transplantation through the portal vein) and Fig. 2F (168 h following BMSC transplantation through the tail vein). In the portal and tail vein transplantation groups, inflammatory cell infiltration was significantly reduced compared with that in the control group. The hepatic lobules were gradually restored. Bile duct hyperplasia in the portal area was also observed. Statistically, the inflammatory activity in the transplantation groups was significantly improved compared with that in the control group (P<0.05; Table V).

### BMSC transplantation inhibits hepatic apoptosis

**Figure 3:**
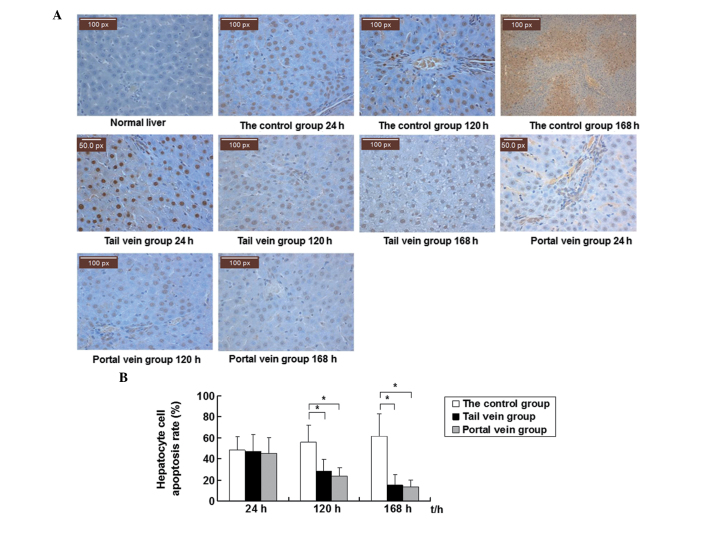
Apoptosis analysis of liver tissue by terminal deoxynucleotidyl transferase dUTP nick end labeling (TUNEL) staining. At 24, 120 and 168 h following bone marrow mesenchymal stem cell (BMSC) transplantation, liver tissue was collected from the normal, control, tail vein and portal vein group rats. Hepatic apoptosis was analyzed by TUNEL staining. (A) Representative TUNEL staining results of normal, control, tail vein and portal vein group liver tissue at the three time points (magnification, ×400). Normal hepatocytes are characterized by blue staining. Apoptotic cells are characterized by brown particles confined within the nucleus. (B) Apoptosis rate of hepatocytes in the control, tail vein and portal vein groups at the three time points. Five fields at high magnification (magnification, ×400) were randomly selected in each slice. The number of apoptotic cells was calculated. The apoptosis rate was calculated as the ratio of TUNEL positive cell number to the total liver cell number/field. ^*^P<0.05, control vs. tail vein and portal vein groups.

Hepatic apoptosis was detected by TUNEL staining. Representative TUNEL staining results are shown in Fig. 3A. In the normal liver tissue, TUNEL negative cells or normal hepatocytes were characterized by blue staining. TUNEL positive cells or the apoptotic cells were characterized by brown particles confined within the nucleus. The apoptotic cells were detected as early as 24 h following transplantation in all three groups. In the control group, the level of apoptosis increased with the progression of liver failure and peaked at 168 h. However, the level of apoptosis decreased at 120 h following BMSC transplantation in the tail and portal vein groups. Normal hepatocytes were observed. At 168 h following BMSC transplantation, the level of apoptosis in the tail and portal vein groups decreased to the lowest level of the experimental period.

Quantitative results are shown in Fig. 3B. Five fields at high magnification (x400) were randomly selected in each slice. The number of apoptotic cells in each field was counted. The apoptosis rate was calculated as the ratio of the TUNEL positive cell number to the total liver cell number per field. At 24 h following transplantation, there were no statistically significant differences in the apoptosis rate among the control (48.00±12.88%), tail vein (47.20±15.71%) and portal vein groups (45.00±14.78%). However, at 120 and 168 h following transplantation, the number of apoptotic cells was reduced in the portal and tail vein groups, whereas in the control group, the number of apoptotic cells was slightly increased compared with the respective values at 24h. The apoptosis rate at 120 h following transplantation in the control, tail vein and portal vein groups was 55.40±16.94, 28.6±11.22 and 23.40±82.95%, respectively. At 168 h following transplantation, the rate of apoptosis in the control, tail vein and portal vein groups was 61.33±21.55%, 15.00±10.39 and 13.17±65.24%, respectively. Statistically, the apoptosis rates in the portal and tail vein groups were significantly lower compared with those in the control group (P<0.05) at 120 h and 168 h following transplantation. There was no significant difference in the rate of apoptosis between the portal and tail vein groups. These data demonstrate that BMSC transplantation is able to inhibit hepatic apoptosis.

### Expression levels of the CD163 and IL-10 proteins decrease in rat liver tissue following BMSC transplantation

**Figure 4:**
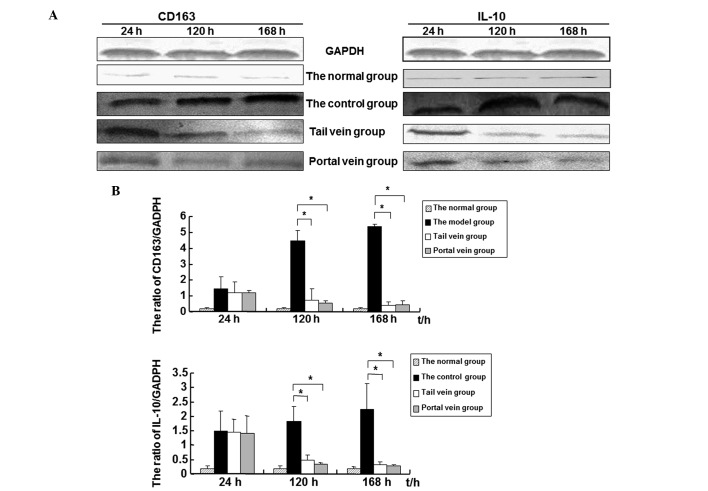
Levels of cluster of differentiation (CD)163 and interleukin (IL)-10 in liver tissue. At 24, 120 and 168 h following bone marrow mesenchymal stem cell (BMSC) transplantation, liver tissue was collected from rats of the normal, control, tail vein and portal vein group. Expression levels of CD163 and IL-10 in the liver tissue were analyzed by western blot analysis. Glyceraldehyde 3-phosphate dehydrogenase (GAPDH) was used as an internal control. (A) Representative western blot analysis results. (B) Quantitative results of the western blot analysis results. Experiments were conducted three times. Data are expressed as mean ± standard deviation. ^*^P<0.05, control vs. tail vein and portal vein groups.

Western blot analysis was performed to detect the expression levels of CD163 and IL-10 in rat liver tissue. Representative western blot analysis results are shown in Fig. 4A and quantitative results are shown in Fig. 4B and Table VI. In the control group, the expression levels of CD163 and IL-10 gradually increased from 24 to 168 h following transplantation. However, in the portal and tail vein groups, the expression levels of CD163 and IL-10 gradually decreased from 24 to 168 h following transplantation. Statistically, there were no significant differences among the three groups in the expression levels of CD163 or IL-10 at 24 h following transplantation. At 120 and 168 h following transplantation, the expression levels of CD163 and IL-10 in the portal and tail vein groups were significantly lower than those in the control group (P<0.05). At the same time, the difference between the portal and tail vein groups in the expression levels of CD163 and IL-10 was not significant. Correlation analysis was carried out between the expression levels of CDl63 and IL-10 in liver tissue. The results revealed that the expression levels of CDl63 were positively correlated with the expression levels of IL-10 at 120 h (r=0.909, P=0.001) and 168 h (r=0.913, P=0.001).

## Discussion

Orthotopic liver transplantation (OLT) is the therapy used as a last resort following the failure of medical management (18,19). Apart from OLT, stem cell transplantation, with the characteristics of simplicity, flexibility and fewer side-effects on the patients, has become another effective means of treating liver failure in recent years (20–22).

The present study revealed that BMSC transplantation may effectively improve liver function, inhibit hepatic apoptosis and alleviate the pathological damage of liver failure in rats. There was no statistically significant difference in the effect of BMSC transplantation between the portal and tail vein groups. This may be due to the spontaneous gathering of stem cells at the damaged site. Cell necrosis in injured tissues releases a series of signaling molecules, triggering the mobilization of bone marrow stem cells to the peripheral blood. The corresponding stem cells bind with specific receptors expressed on the injured tissues and attach to the damaged site in order to repair the injured tissues (23,24). However, this may also be caused by the short detection time of the present study, since the effect of BMSC transplantation was not analyzed for longer than 168 h. Thus, whether the effect of portal vein transplantation differs to that of tail vein transplantation requires further investigation.

Previous studies have demonstrated that BMSCs promote hepatic regeneration, inhibit inflammation, apoptosis and hepatic stellate cell activation, degrade the redundant extracellular matrix and improve liver fibrosis via the paracrine production of cytokines and growth factors (25–27). In the current study, the apoptosis rate of hepatocytes following transplantation was significantly decreased, indicating that BMSCs were able to reduce the inflammatory response in the liver and inhibit hepatic apoptosis.

Studies have demonstrated that there are varying degrees of endotoxemia and inflammatory cascades in AHF, leading to changes in the levels of cytokines (28). Endotoxemia, macrophage overactivation and proinflammatory cytokines may induce the expression of CD163. CD163 activates monocyte and macrophage cells *in vivo* and mediates heme oxygenase-1 synthesis. Heme oxygenase-1 exerts anti-inflammatory, antioxidant and anti-apoptotic effects on the body. Degradation of heme by heme oxygenases results in the production of CO, iron and biliverdin (29,30). Previous studies have demonstrated that IL-10 and the macrophage colony-stimulating factor (M-CSF) may induce the differentiation of monocytes into macrophages, which may further upregulate CD163 mRNA and protein expression. However, CD163 may also induce macrophages to secrete anti-inflammatory cytokines, including IL-10 (31,32). A study has identified that CD163 is a receptor of the tumor necrosis factor (TNF)-like weak inducer of apoptosis (TWEAK) cytokine; by binding with TWEAK, CD163 may induce the endocytosis of TWEAK and inhibit the proapoptotic function of TWEAK (33). In the current study, the levels of CD163 and IL-10 in the serum and liver tissue were detected by ELISA and western blot analysis. It was observed that the expression levels of CDl63 were correlated with the expression levels of IL-10. The levels of CD163 and IL-10 increased with the increase in the level of apoptosis, indicating an anti-apoptotic role of CD163 and IL-10 in the liver.

Liver failure is a severe disease with a high mortality rate. Thus, evaluating the patient condition and prognosis objectively, effectively and in a timely manner is of great importance. The current liver failure prognosis assessment system is widely used in clinical practice. However, in order to improve its accuracy, new evaluation indices are required. In the present study, following BMSC transplantation, the levels of ALT, AST, DBIL and IBIL significantly decreased with time whereas the level of ALB significantly increased. These data suggest that liver function was improved by BMSC transplantation. Furthermore, with the elevation of the levels of ALT and AST in the plasma, the levels of CDl63 and IL-10 in the serum and liver tissue of rats with AHF were significantly increased in the control group compared with those in normal mice. These levels of CDl63 and IL-10 were significantly higher than those in the BMSC transplantation group. Furthermore, the levels of CDl63 and IL-10 were significantly correlated with the levels of ALT and AST, which reflected the sharp deterioration in liver function and disease severity. These results suggest that CDl63 and IL-10 play important roles in the pathogenesis of AHF and that CDl63 and IL-10 may be used as sensitive serum marker proteins for the diagnosis and prognosis of AHF. Following BMSC transplantation, the levels of CDl63 and IL-10 in the serum and liver tissue decreased and liver function was gradually restored. These data indicate that BMSC transplantation may improve the immune status of AHF model rats and that BMSC transplantation had a protective effect on rats with AHF. Thus, through downregulating the levels of CDl63 and IL-10, BMSC transplantation promoted the inflammatory and anti-inflammatory cytokines to reach a new equilibrium. This may be one of the mechanisms by which BMSCs exert their therapeutic functions on AHF. In summary, the present results demonstrated the therapeutic effect of BMSCs on AHF. In the future, CD163 and IL-10 may be used as sensitive serum prognosis indicators in the early assessment of patients undergoing liver transplantation.

In summary, BMSCs are of great scientific and clinical value for the treatment of AHF. However, further studies are necessary to explore the potential of BMSC differentiation and treatment mechanisms. Further study is also required to observe the changes in liver function and cytokines for longer than 168 h following liver BMSC transplantation in order to clarify the treatment mechanisms and investigate the effect of BMSC transplantation.

## Figures and Tables

**Table I:** Serum levels of ALT and AST in each group at the three time points (IU/l).

	24 h		120 h		168 h	
			
Groups	ALT	AST	ALT	AST	ALT	AST
Normal	24.00±3.74	29.50±6.35	26.50±3.87	30.75±4.29	27.00±2.45	32.50±4.20
Control	140.20±44.42	182.00±79.78	152.00±72.60	188.00±90.24	155.40±34.81	193.20±55.54
Tail vein	134.60±58.08	179.20±86.68	95.33±12.19^a^	113.33±18.71^a^	71.50±16.36^a^	95.67±13.84^a^
Portal vein	131.00±54.47	173.50±93.10	76.50±7.29^a^	99.17±14.28^a^	49.33±13.50^a^	71.50±24.56^a^

Data are expressed as mean ± standard deviation.

a P<0.05 when compared with the control group.

DBIL, direct bilirubin; IBIL, indirect bilirubin.

**Table II:** Serum levels of DBIL and TBIL in each group at the three time points (IU/l).

	24 h		120 h		168 h	
			
Groups	DBIL	IBIL	DBIL	IBIL	DBIL	IBIL
Normal	1.78±0.40	1.9±0.50	3.15±1.57	2.38±1.03	1.92±1.07	3.38±1.48
Control	12.08±5.90	13.4±2.60	30.46±9.54	29.64±8.97	30.83±4.98	24.68±6.57
Tail vein	10.92±5.21	10.38±7.44	6.82±1.15^a^	7.02±1.84^a^	5.73±4.35^a^	5.57±2.28^a^
Portal vein	10.94±5.01	7.00±3.93	7.12±2.83^a^	4.98±2.69^a^	6.08±4.35^a^	4.07±2.00^a^

Data are expressed as mean ± standard deviation.

a P<0.05 compared with the control group.

ALB, albumin; DBIL, direct bilirubin; IBIL, indirect bilirubin.

**Table III:** Serum levels of ALB in each group at the three time points (IU/l).

Groups	24 h	120 h	168 h
Normal	40.00±3.92	39.5±2.89	42.75±6.95
Control	24.78±1.77	23.86±1.69	21.63±4.62
Tail vein	23.80±3.18	24.82±4.08	25.87±3.52^a^
Portal vein	25.04±2.70	25.66±2.59	25.97±4.68^a^

Data are expressed as mean ± standard deviation.

a P<0.05 compared with the control group.

ALB, albumin.

**Table IV:** Serum levels of CDl63 and IL-10 in each group at the three time points (pg/ml).

	24 h		120 h		168 h	
			
Groups	CDl63	IL-10	CDl63	IL-10	CDl63	IL-10
Normal	6.00±5.38	11.81±4.65	6.17±7.09	11.06±1.36	5.66±4.95	11.90±6.69
Control	32.49±4.73	31.10±9.66	34.28±7.30	35.23±9.60	37.04±7.50	37.52±15.67
Tail vein	32.04±7.04	30.39±4.37	23.80±6.85^a^	23.72±5.28^a^	15.47±8.81^a^	20.74±5.00^a^
Portal vein	31.32±8.27	30.20±13.21	14.73±5.83^a^	19.01±2.04^a^	9.43±6.08^a^	13.20±2.59^a^

Data are expressed as mean ± standard deviation.

a P<0.05 compared with the control group.

CD163, cluster of differentiation 163; IL-10, interleukin 10.

**Table V:** Inflammatory activity of the liver tissues in each group at the three time points.

Groups	24 h	120 h	168 h
Control	43.20±10.73	46.40±14.31	46.67±12.82
Tail vein	41.60±17.34	22.00±16.84^a^	18.33±8.78^a^
Portal vein	38.20±22.54	20.60±7.60^a^	12.67±8.78^a^

Data are expressed as mean ± standard deviation.

a F=9.548, P<0.05 when compared with the control group.

**Table VI:** Expression levels of CDl63 and IL-10 protein in liver tissue (relative to GAPDH).

	24 h		120 h		168 h	
			
Groups	CDl63	IL-10	CDl63	IL-10	CDl63	IL-10
Normal	0.20±0.64	0.19±0.10	0.20±0.56	0.18±0.10	0.19±0.09	0.18±0.08
Control	1.47±0.74	1.50±0.70	4.48±0.68	1.82±0.52	5.37±0.17	2.27±0.87
Tail vein	1.21±0.68	1.46±0.45	0.75±0.72^a^	0.49±0.17^a^	0.41±0.23^a^	0.32±0.10^a^
Portal vein	1.18±0.17	1.40±0.61	0.54±0.16^a^	0.35±0.56^a^	0.47±0.23^a^	0.28±0.56^a^

Data are expressed as mean ± standard deviation.

a P<0.05 compared with the control group.

CD163, cluster of differentiation 163; IL-10, interleukin 10.
